# 非小细胞肺癌*EGFR*、*KRAS*、*ALK*基因突变与不同转移器官分布的相关性研究进展

**DOI:** 10.3779/j.issn.1009-3419.2018.07.06

**Published:** 2018-07-20

**Authors:** 歌 高, 立力 邓

**Affiliations:** 150086 哈尔滨，哈尔滨医科大学附属第二医院肿瘤内科 Department of Oncology, The Second Affiliated Hospital of Harbin Medical University, Harbin 150086, Chinaa

**Keywords:** 转移, 肺肿瘤, EGFR, ALK, KRAS, Metastasis, Lung neoplasms, EGFR, ALK, KRAS

## Abstract

肺癌是我国恶性肿瘤的首位死亡疾病，据统计大约57%的肺癌患者就诊时已经出现了远处转移，临床预后较差。抗肺癌转移是当前治疗晚期转移性肺癌的新方向和思路。既往研究表明肿瘤的生物学改变在一定程度上能够影响肿瘤的转移行为和侵袭扩散模式，而目前的基础及临床研究尚未阐明导致肺癌相关信号转导途径中发生特异性器官转移的分子机制，有关驱动基因突变与器官转移之间相关性的研究也较为罕见。本篇综述旨在对近几年有关非小细胞肺癌表皮生长因子受体（epidermal growth factor receptor, *EGFR*）、间变性淋巴瘤激酶（anaplastic lymphoma kinase, *ALK*）、Kristen鼠肉瘤病毒原癌基因同源体（V-Ki-ras2 Kirsten rat sarcoma viral oncogene homologue, *KRAS*）驱动基因表达的特点以及与转移器官分布之间相关性的文献进行小结。

## Abstract (en)

Lung cancer is the leading cause of morbidity and mortality of malignant diseases in China. Approximately 57% lung cancer patients harbored distant metastases at initial diagnosis which is relevant to poor outcomes. The research strategy of anti-lung cancer metastasis now has became the new treatment directions and thoughts for lung cancer treatment. Previous studies have shown that changes in the corresponding driving genes on different signaling pathways may be related to the transfer of different organs, and the biological alteration of tumor to some extent can affect the metastatic behavior and metastatic pattern of tumor. However, current clinical and basic studies have not elucidated the molecular mechanism of the specific distant organ metastasis in the pathway of lung cancer related signal transduction, clinical research on the correlation between gene mutation and organ transfer specificity is also relatively rare. This review aims to summarize the characteristics of the expression of epidermal growth factor receptor (EGFR), anaplastic lymphoma kinase (ALK), V-Ki-ras2 Kirsten rat sarcoma viral oncogene homologue (KRAS) in non-small cell lung cancer, and the correlation between the distribution of metastatic organs.

肺癌是居于我国第一位的恶性肿瘤致死性疾病，受限于早期诊断的局限，以及高转移性的生物学特点，大部分肺癌患者就诊时已经出现了远处转移，失去早期手术治疗的机会^[1]^。肺癌常见远处转移部位包括脑、骨、肝脏、肾上腺和肺^[2]^。虽然局部治疗和化疗甚至靶向治疗能够在一定程度上改善转移性病变的治疗应答率，但是不同器官转移的肺癌患者预后差异仍然很大，目前针对不同器官转移进行特异性治疗的研究也较罕见。随着以奥西替尼为代表的第三代靶向药物的应用，推动了我国肺癌诊疗进入精准治疗3.0时代。研究者对肺癌分子生物学的研究越来越深入，逐渐认识到肺癌远处转移是一个涉及多基因调控、多信号传导通路共同参与的复杂过程^[3]^。不同信号通路上相应驱动基因的改变在一定程度上能够影响肿瘤的转移行为和侵袭扩散模式。但是目前关于驱动基因突变与器官转移之间的相关性研究较为罕见，尚无统一定论。进一步认识非小细胞肺癌（non-small cell lung cancer, NSCLC）驱动基因突变的临床特征以及和特异性器官转移模式之间的相关性或许能够提供一些更有效的治疗策略以提高晚期转移性肺癌患者的预后，最大限度地改善患者生活质量，延长生存期。本篇综述旨在对近几年国内外关于NSCLC表皮生长因子受体（epidermal growth factor receptor, *EGFR*）、间变性淋巴瘤激酶（anaplastic lymphoma kinase, *ALK*）、Kristen鼠肉瘤病毒原癌基因同源体（V-Ki-ras2 Kirsten rat sarcoma viral oncogene homologue, *KRAS*）驱动基因表达的特点以及与转移器官分布之间相关性的文献进行小结。

## 1 *EGFR*基因与器官转移

*EGFR*是NSCLC最主要的驱动基因。在东亚人群中突变率高达40%-50%，而西方人群中突变率为10%-20%^[4]^。*EGFR*基因突变主要发生在18号-21号外显子，不同位点的突变在临床特点、肿瘤扩散路径等方面具有一定的差异。既往研究已经证实EGFR信号转导通路在肿瘤发生发展过程中起着十分重要的作用^[3]^。然而目前关于EGFR信号通路与NSCLC转移扩散之间的关系仍然不明确，因此，我们非常有必要探索*EGFR*基因突变患者的转移特点，以期为临床进一步筛查以及治疗提供依据。

### 1.1 *EGFR*基因与脑转移

肺癌是最容易出现脑转移的原发肿瘤，晚期转移性肺癌中，脑转移比例高达20%-40%，脑转移已经成了晚期肺癌患者致死的主要原因^[5]^。并且，近几年脑转移的发生率有逐渐增加的趋势，一部分归因于脑成像技术的改善使得脑转移灶的诊断率提高，另一方面由于近几年对原发肿瘤治疗手段的不断提升，如颅内手术切除、全脑放射性照射（whole brain radiation therapy, WBRT）、立体定位放射手术（stereotactic radiosurgery, SRS）、化疗、靶向治疗等，患者生存期得到改善，在一定程度上也增加了脑转移的发生率。许多临床研究都试图探索NSCLC脑转移的预测因素，包括肿瘤标志物水平^[6]^、原发肿瘤大小、淋巴结分期等。近几年，一些研究也报道了脑转移与*EGFR*基因状态之间的相关性。

Guan等^[7]^回顾性分析了401例NSCLC患者初诊时*EGFR*突变状态与器官转移的关系，所有患者初诊时均接受正电子发射计算机断层显像与计算机断层扫描技术（positron emission tomography computed tomography, PET/CT）检查。研究发现*EGFR*基因突变的NSCLC患者无论是在数量上还是频率上都较EGFR野生型的患者更容易出现脑转移。国内外其他多项研究^[8-11]^均支持这一结论。此外，Li等^[9]^和Takano等^[10]^均发现携带EGFR 19号外显子缺失性突变的患者与其他突变亚型相比脑转移发生率更高，并且特征性的脑转移模式是出现多发粟粒状脑转移瘤，而脑水肿较轻。而携带21号外显子L858R点突变的患者则更容易出现尾状核、小脑、颞叶部位的转移。

*EGFR*基因促进脑转移的具体机制尚不明确。Breindel等^[12]^报道了EGFR信号通路通过丝裂原活化蛋白激酶激活下游*MET*基因从而促进NSCLC发生脑转移。表明了其他罕见基因突变如*MET*基因突变或*EGFR*基因下游信号转导通路的改变在脑转移瘤发生中的潜在作用。此外，非小细胞肺癌*EGFR*基因变异能够促进信号传导与转录激活因子（signal transducers and activators of transcription 3, STAT3）的激活，而近期Singh等^[13]^发现STAT3信号途径能够通过抑制miR-21的表达导致肺癌细胞出现脑转移。STAT3或许是未来治疗肺癌脑转移的潜在治疗靶点。虽然这些研究探讨了一些关于*EGFR*基因突变的NSCLC出现脑转移风险增加的可能的机制，但是仍然需要进一步深入研究明确*EGFR*基因在分子水平上促进脑转移的确切作用。

### 1.2 *EGFR*基因与骨转移

除了脑转移外，骨转移同样给NSCLC患者带来了不良预后。肺癌骨转移多表现为溶骨性破坏，出现骨密度降低、病理性骨折、骨痛等严重并发症，降低患者生存质量。并且骨转移患者往往肿瘤恶性程度较高，临床预后较差。

Fujimoto等^[14]^对277例Ⅳ期肺腺癌患者进行回顾性分析，所有患者均未行肺癌相关治疗，包括手术治疗、放化疗、靶向治疗等，结果发现*EGFR*突变型的肺腺癌患者与野生型患者相比骨转移灶数量更多（*P*=0.035），但骨转移发生率并未发现统计学差异（*P*=0.076）。生存数据分析结果显示，*EGFR*基因突变的患者，伴或不伴骨转移总生存期（overall survival, OS）具有明显统计学差异（中位OS 22.7个月*vs* 35.2个月，*P*=0.002）。说明骨转移对于*EGFR*突变型的晚期肺腺癌患者来说是一个不良预后的独立预测因素。此外，Li等^[9]^学者的大样本研究同样显示*EGFR*基因突变状态与骨转移发生率之间不具有统计学差异。

然而，由于遗传异质性的存在，同一患者原发灶与转移灶肿瘤细胞可能存在不同的基因突变状态。而上述研究中绝大部分患者基因突变状态往往是通过原发肿瘤活检组织或手术组织确定。来自法国的POUMOS-TEC研究^[15]^共入组49例晚期肺腺癌骨转移的患者，通过手术或者CT引导下穿刺获取骨转移灶活检组织进行*EGFR*、*KRAS*、*ALK*等肺癌驱动基因检测。结果发现超过50%的患者骨转移组织中具有至少一种基因突变，其中*EGFR*突变频率为14%，高于法国晚期肺腺癌*EGFR*基因平均突变率。这意味着*EGFR*突变的肺腺癌肿瘤细胞可能对骨组织的亲和力更高，更容易出现骨转移。

目前的研究尚不能完全阐明*EGFR*基因通路与骨微环境之间存在的特定的相互作用。一种假设是在骨转移的发生发展过程中，有一些破骨相关因子在骨吸收过程中从骨基质中被释放出来，例如转化生长因子-β（transforming growth factor-β, TGF-β），这些破骨相关因子增强了肿瘤细胞的生长以及上皮间质转化（epithelial-mesenchymal transition, EMT），导致破骨作用，促进骨转移^[16]^。其次，一些研究认为EGFR信号转导系统能够通过下调一些肿瘤相关因子如血管内皮生长因子-A（vascular endothelial growth factor-A, VEGF-A）、血管内皮生长因子受体-1（vascular endothelial growth factor receptor-1, VEGFR-1）以及肿瘤坏死因子-α（tumor necrosis factor-α, TNF-α）的表达，从而营造一个能促进原发肿瘤组织发生侵袭的环境^[17]^。由于骨转移与不良预后及缩短患者生存时间相关，且越来越多的临床药物可有效治疗和缓解骨转移。因此，越早发现骨转移就越能较早地采取干预措施，提髙患者的预后及生存质量。

### 1.3 *EGFR*基因与内脏转移

肺癌内脏转移如肝、肺、肾上腺等转移相对于脑转移和骨转移发生率较低，目前有关*EGFR*基因表达状态与内脏转移相关性的文献报道较少见。一项来自我国的1, 063例患者的大样本回顾性分析^[9]^显示，初诊时*EGFR*突变状态与远处转移之间的相关性具有统计学意义。亚组分析显示携带*EGFR*基因19外显子缺失性突变与肺转移密切相关（OR=1.587, 95%CI: 1.065-2.346, *P*=0.023）。而21号外显子突变更容易出现肝转移（OR=1.987, 95%CI: 1.094-3.067, *P*=0.024）。此外，Doebele等^[18]^研究同样发现*EGFR*基因与肝转移密切相关（*P*=0.006）。

另一项来自日本一项小样本研究^[19]^显示*EGFR*基因突变的肺腺癌患者容易出现随机的弥散性肺内转移，而相比之下，*EGFR*野生型的肺腺癌患者肺内转移却较为罕见。研究者认为*EGFR*突变型出现这种弥散性肺内转移的模式意味着血源性转移与肿瘤血管生成密切相关。EGFR信号通路在肿瘤恶化、转移过程中发挥着重要作用。由于EGFR信号通路能够调节肿瘤细胞中各种不同的血管生长因子的合成与分泌，例如血管内皮生长因子、白介素-8、纤维细胞生长因子等。因此*EGFR*突变率较高的肿瘤如NSCLC、甲状腺癌，则倾向于发展血管生成性转移，例如弥散性肺转移。

然而，Yasunori等^[20]^日本学者却发现*EGFR*基因突变状态与肺、脑或肝转移并没有明确的相关性，但是*EGFR*基因突变的患者与EGFR野生型的患者相比出现肺转移时病灶数目更多，尤其是19号外显子缺失性突变亚型的患者。另外，Fujimoto等^[14]^对277例Ⅳ期肺腺癌患者进行回顾性分析同样未发现*EGFR*基因突变状态与肝转移、肺转移之间的相关性。

## 2 *ALK*基因与器官转移

*ALK*基因是一种受体酪氨酸激酶，能参与调控细胞增殖的信号通路。在NSCLC中*ALK*基因最常见的突变方式是与棘皮动物微管蛋白4（echinoderm microtubule-associated protein like 4, EML4）形成*EML4-ALK*融合基因。据统计，*ALK*基因重排出现在3%-5%的NSCLC中^[21]^，与*EGFR*、*KRAS*基因相比，突变率较低。近期一些研究同样发现*ALK*基因突变的肺腺癌具有特定的转移扩散模式。

### 2.1 *ALK*基因与淋巴结转移

近期日本学者发表了一项关于*ALK*基因重排的晚期NSCLC患者影像学特征的小样本研究^[22]^，结果显示CT表现为小体积、实性结节、出现淋巴结转移或者胸腔积液的患者更容易出现*ALK*基因重排。说明*ALK*基因突变的NSCLC可能有肿瘤浸润到周围的支气管血管鞘或向局部的淋巴管扩张的趋势。韩国Choi等^[23]^研究纳入331例肺腺癌患者，结果显示*ALK*基因突变的肺腺癌患者与*EGFR*基因突变的患者（*P* < 0.01）以及ALK和EGFR均为野生型的患者（*P* < 0.01）相比更容易出现淋巴结转移。Tian等^[24]^的回顾性研究同样发现*ALK*基因重排的肺腺癌患者与淋巴结转移密切相关，并且远处淋巴结转移多发生于腹腔，少数为腋窝淋巴结转移。然而，与上述研究相反，Doebele等^[18]^并未发现*ALK*基因与淋巴结转移的相关性，无论是肺内淋巴结（*P*=0.09）还是肺外淋巴结（*P*=0.07），差异均无统计学意义。

### 2.2 *ALK*基因与胸膜转移

国内Tian等^[24]^研究发现，初诊Ⅳ期伴*ALK*基因突变的肺腺癌患者最常见的远处转移部位是胸膜转移，表现为胸膜结节或者恶性胸腔积液。另一项对*ALK*融合基因阳性的52例NSCLC患者的研究中^[25]^，恶性胸腔积液发生率为15.4%，与传统的非选择性人群相比发生率较高，意味着*ALK*基因突变与胸膜转移可能具有相关性。Doebele等^[18]^研究发现，与*EGFR*、*ALK*和*KRAS*基因为野生型的患者相比，*ALK*基因突变与胸膜扩散呈高度密切相关（*P*=0.000, 2）。此外，该研究发现*ALK*基因融合与心包扩散（*P*=0.02）和肝转移（*P*=0.003）同样密切相关。同时，*ALK*基因阳性的亚组常表现为多发远处转移（平均值=3.6个转移灶，*P* < 0.000, 1），意味着*ALK*基因突变的患者预后相对更差。

然而，国内Chen等^[26]^的大样本研究并未发现*ALK*基因重排与胸膜转移的相关性，研究发现*ALK*基因重排的晚期肺癌患者在基线确诊时（6.2% *vs* 26.9%; *P* < 0.001）以及治疗过程中（3.1% *vs* 10.3%; *P*=0.031）恶性胸水的发生率均明显低于ALK/EGFR双阴性的患者。出现不一致结论的原因可能是由于样本量的差异以及回顾性研究的偏倚。未来多中心的前瞻性研究可能会发现ALK阳性的病人是否有更高的胸膜转移率。

### 2.3 *ALK*基因与脑转移

*ALK*基因与脑转移的关系目前并没有足够的研究证据支持。一项我国的大样本、单中心的真实世界研究^[26]^探讨了*ALK*基因重排的晚期NSCLC患者临床病理特征及治疗预后，研究发现无论是在基线确诊时（26.5% *vs* 16.5%, *P*=0.038）还是在治疗过程中（25.8% *vs* 11.9%, *P*=0.003），*ALK*基因阳性组脑转移的发生率均高于ALK/EGFR双阴性组。此外，亚组分析显示脑转移数量在两组之间同样具有差异。基线确诊时，*ALK*基因阳性组更容易出现单发脑转移瘤（11.3% *vs* 4.6%; *P*=0.033），而在治疗过程中，*ALK*基因阳性的患者与ALK/EGFR双阴性的患者相比，多发性脑转移瘤的发生率较高（24.7% *vs* 8.2%, *P* < 0.001）。

脑转移是肺癌患者最常见的转移部位，预后差，死亡率高。基于ALK阳性的患者更倾向于出现脑转移，是否意味着这类患者更倾向于通过血源性传播来形成远处器官的转移。遗憾的是，目前尚未发现有文献探讨了相关分子机制，但是也提醒了临床医生对于ALK阳性的患者，脑转移的预防与常规监测应贯穿治疗全过程。

## 3 *KRAS*基因与器官转移

*KRAS*基因是RAS家族的一个原癌基因。*KRAS*基因突变与肺癌、结直肠癌、胰腺癌密切相关。在NSCLC尤其是肺腺癌中，*KRAS*基因突变率在东西方人种中具有较大差异，*KRAS*基因是西方人种最常见的突变基因，突变率接近30%，而在亚洲人群中突变率仅为5%^[27]^。*KRAS*基因位于EGFR信号通路下游，在EGFR信号传导通路中起着“开关”的重要作用，一旦*KRAS*基因持续活化突变，则多种分裂、增殖因子被持续激活，针对EGFR多种靶向药物往往疗效较差^[28]^。尽管近十年来关于*KRAS*基因与预后关系的研究广泛，但多以结直肠癌研究为主。多项研究^[29, 30]^指出，*KRAS*基因突变的结直肠癌患者预后较差，发生远处转移如肺、骨、脑转移的概率明显增加。但是目前关于*KRAS*基因与肺癌转移器官特异性之间相关性研究十分罕见。

### 3.1 *KRAS*基因与脑转移

国外Wilkerson等^[31]^研究显示同时具有*KRAS*基因突变以及*LKB1*基因低拷贝数的肺腺癌患者与脑转移的发生密切相关（*P*=0.007），但是导致脑转移的具体机制尚不明确。*LKB1*是一种重要的抑癌基因，据统计大约30%的NSCLC患者*LKB1*基因呈失活状态。*LKB1*基因通过编码一种在细胞中广泛表达的丝氨酸/苏氨酸蛋白激酶，通过腺苷酸活化蛋白激酶（adenosine 5' -monophosphate (AMP)-activated protein kinase, AMPK）级联反应调节细胞新陈代谢。*LKB1*基因缺失能抑制AMPK，通过提高细胞生长正调节因子mTORC1的活性，导致细胞发生恶性增殖。小鼠黑素细胞*LKB1*基因失活以及*KRAS*基因突变可以导致100%外显率的高转移性黑色素瘤，表明癌细胞转移可能是*LKB1*基因失活伴*RAS*基因突变同时异常所导致的不良后果^[32]^。

### 3.2 *KRAS*基因与其他器官转移

2016年德国一项回顾性研究^[33]^显示，*KRAS*基因突变患者更容易出现肺内转移，而肝转移、胸膜转移发生率较低。该结论与*KRAS*基因与多项结直肠癌研究结果^[34, 35]^相似。此外，目前虽尚未有研究发现*KRAS*基因与骨转移的相关性，但是国外Confavreux和Bittner等^[15, 36]^发现*KRAS*基因突变的患者中，出现骨转移预后最差。表明了*KRAS*基因突变对于骨转移的肺癌患者来说可能是一个预后的独立预测指标。

## 4 小结

尽管近几年肺癌的基因治疗不断改进，在一定程度上提高了患者的生存率，但是目前肺癌在全球范围内仍是死亡率最高的疾病之一，复发和转移仍然是致死率的主要原因。尤其是对于靶向治疗的患者来说，生存期得到了提高，但是进展后出现器官转移的概率也大大增加。因此，抗肺癌转移成为当前治疗晚期转移性肺癌的新方向和思路。然而，目前的基础及临床研究尚未阐明导致肺癌相关信号转导途径中发生特异性远处器官转移的分子机制，驱动基因突变与器官转移之间是否具有相关性争论较大。本篇综述总结了近几年国内外相关文献（表 1），*EGFR*突变的NSCLC患者出现脑转移、骨转移的概率明显增加，并且骨转移数量更多。内脏转移中肺转移、肝转移与*EGFR*基因突变相关性争议较大，有待进一步大样本数据支持。此外，携带19号外显子缺失性突变的患者脑转移发生率较其他亚型更高，而21号外显子突变可能与肝转移具有相关性。*ALK*基因重排的患者则更容易出现淋巴结、胸膜和脑转移。*KRAS*基因突变的患者肺内转移发生率较高，而肝转移、胸膜转移发生率较低。此外，*KRAS*基因突变同样与脑转移的发生密切相关。

**1:** 非小细胞肺癌*EGFR*、*ALK*、*KRAS*突变的不同转移途径及分子机制 Different metastatic pathways and molecular mechanisms of *EGFR*, *ALK* and *KRAS* mutations in non-small cell lung cancer (NSCLC)

Genes	Metastatic sites	Molecular mechanism
*EGFR*	Brain^[7-11]^	*MET* gene activation^[12]^;STAT3 signaling pathway activation^[13]^;
	Bone^[14, 15]^	Increased release of osteoclastic related factors^[16]^;Down-regulation of tumor related factors VEGF-A，VEGFR-1，TNF-α expressions^[17]^;
	Lung^[9, 19]^	EGFR signaling pathway regulates the synthesis and secretion of vascular endothelial growth factor increased^[19]^
	Liver^[9, 18]^	-
*ALK*	Lymph node^[22-24]^	-
	Pleura^[18, 24, 25]^	-
	Brain^[26]^	-
*KRAS*	Lung^[33]^	-
	Brain ^[31]^	*LKB1* gene inactivation^[31, 32]^
EGFR: epidermal growth factor receptor; ALK: anaplastic lymphoma kinase; KRAS: V-Ki-ras2 Kirsten rat sarcoma viral oncogene homologue.		

远处转移是影响肺癌病程和预后的主要因素，如何预测转移方向，从而针对高危人群进行有效地预防和治疗，是接下来需要解决的问题。越来越多的临床数据已经表明NSCLC *EGFR*、*ALK*、*KRAS*驱动基因的突变具有特定的转移途径，肿瘤的生物学改变在一定程度上能够调节癌细胞转移扩散的模式。并且，有研究发现从细胞系中提取出来的基因表达标记能够重复的转移到一个特定的器官部位，表明这个转移过程是经过程序化控制的，而不仅仅是一个随机的结果^[37]^。然而，由于本综述大部分文献研究样本量较少，多为回顾性分析，并且大部分研究并未对原发肿瘤和转移标本进行配对，关于基因表达与器官转移特异性的结论并不是绝对的。其次，由于转移位置在治疗的全过程会发生变化，本综述查阅的文献并未分别对基线确诊时转移特征与全病程转移特征进行总结分析，因此进一步开展多中心前瞻性研究十分重要。此外，使用携带不同驱动基因突变状态的NSCLC肿瘤细胞动物移植实验模拟明确器官转移分子机制仍然十分必要。

临床上我们虽然提倡对所有NSCLC患者进行肺癌相关分子标记检测，但明确驱动基因突变与不同转移扩散模式之间的联系可以为筛选分子检测人群提供额外的临床提示。同时能够在疾病诊断、治疗策略上提供更有价值的信息。此外，明确晚期肺癌发生转移的分子机制及信号调节途径，可以为未来研发可能抑制或逆转肺癌转移的分子靶向药物提供新的线索。
